# Adenosine stress perfusion CMR in young children: assessment of optimal imaging parameters

**DOI:** 10.1186/1532-429X-15-S1-P298

**Published:** 2013-01-30

**Authors:** Michael J Campbell, Piers Barker, Stephen Darty, Raymond J Kim

**Affiliations:** 1 Duke Cardiovascular Magnetic Resonance Center, Duke Unviersity Hospital, Durham, NC, USA

## Background

Adenosine stress perfusion CMR is commonly used for assessing coronary artery disease (CAD) in adults. CAD is uncommon in children, but does occur. There is limited experience performing adenosine stress perfusion CMR in young children. Performing stress perfusion CMR in young children presents a number of technical imaging challenges.

Specific Aim: Evaluate image quality and optimal imaging parameters in young children undergoing adenosine stress perfusion CMR.

## Methods

Consecutive patients, who completed clinically ordered CMR adenosine stress perfusion and were </= 5yo or </= 25kg, were enrolled. General anesthesia was utilized in all. All studies were performed on a 1.5T Siemens Avanto system. Adenosine stress perfusion was performed with administration of adenosine (140 μg/kg/min) for 2-4 minutes and gadolinium (0.1 mmol/kg). A prospectively gated saturation recovery turbo flash image was acquired. Imaging parameters were selected by the technician at the time of each scan to optimize image quality. Images were retrospectively reviewed to assess diagnostic quality. The following imaging parameters were recorded: field of view (FOV)-read, FOV-phase, matrix, inversion time, slice thickness and spatial resolution.

## Results

7 patients were enrolled. Demographic information is listed in table 1. All completed stress perfusion CMR without adverse events. All had images of diagnostic quality. 2 patients had perfusion defects consistent with inducible ischemia. One underwent coronary angiography (1 lost to follow-up), and had CAD consistent with stress perfusion CMR. One patient with negative stress perfusion CMR underwent coronary angiography and did not have CAD.

**Table 1:** Patient Demographics

Patient	Age (yr)	Weight (kg)	Diagnosis
1	1	8.6	Transposition of the great arteries s/p arterial switch
2	2	14.6	Transposition of the great arteries s/p arterial switch
3	4	22.3	Pulmonary atresia/intact ventricular septum s/p Fontan
4	5	20.7	Anomalous origin of left coronary artery from pulmonary artery s/p repair
5	5	18.5	Transposition of the great arteries s/p arterial switch
6	5	22.5	Double outlet right ventricle s/p ventricular septal defect closure and pulmonary valvotomy
7	5	19.4	Transposition of the great arteries s/p arterial switch

Imaging parameters are listed in table 2. The average FOV-read was 237mm (180-300mm), FOV-phase was 175mm (135-239mm), matrix (frequency) was 198mm (160-300mm) and matrix (phase) was 146mm (112-139mm). The average inversion time was 136msec (100-200 msec). Slice thickness was 7-8mm. The average spatial resolution in both the read and phase direction was 1.2mm (0.9-1.6mm), and the average voxel size was 1.2 x 1.2 x 7.5mm.

**Table 2:** Stress Perfusion CMR: Imaging Parameters

Patient	Age (yr)	Weight (kg)	FOV-read (mm)	FOV-phase (mm)	Matrix-frequency (mm)	Matrix-read (mm)	TI (msec)	Slice thickness (mm)	Spatial resolution-read (mm)	Spatial resolution-phase (mm)
1	1	8.6	220	154	160	112	110	8	1.4	1.4

2	2	14.6	180	135	192	144	180	7	0.9	0.9

3	4	22.3	300	239	300	239	130	8	1.0	1.0

4	5	20.7	200	139	176	122	120	8	1.1	1.1

5	5	18.5	220	150	208	142	200	7	1.1	1.1

6	5	22.5	260	192	160	118	100	8	1.6	1.6

7	5	19.4	280	219	192	150	115	8	1.5	1.5

FOV=Field of view, TI=Inversion time

## Conclusions

CMR adenosine stress perfusion can be performed in young children and images of diagnostic quality acquired. Young children present a number of technical challenges that can be overcome by appropriately adapting imaging parameters to the individual patient size.

## Funding

None.

